# Costs and cost-effectiveness of vector control in Eritrea using insecticide-treated bed nets

**DOI:** 10.1186/1475-2875-8-51

**Published:** 2009-03-30

**Authors:** Joshua O Yukich, Mehari Zerom, Tewolde Ghebremeskel, Fabrizio Tediosi, Christian Lengeler

**Affiliations:** 1 Swiss Tropical Institute, P.O. Box, 4002 Basel, Switzerland; 2 National Malaria Control Programme, Asmara, Eritrea; 3 Centre for Research on Health and Social Care Management, Università Bocconi, Milan, Italy

## Abstract

**Background:**

While insecticide-treated nets (ITNs) are a recognized effective method for preventing malaria, there has been an extensive debate in recent years about the best large-scale implementation strategy. Implementation costs and cost-effectiveness are important elements to consider when planning ITN programmes, but so far little information on these aspects is available from national programmes.

**Methods:**

This study uses a standardized methodology, as part of a larger comparative study, to collect cost data and cost-effectiveness estimates from a large programme providing ITNs at the community level and ante-natal care facilities in Eritrea. This is a unique model of ITN implementation fully integrated into the public health system.

**Results:**

Base case analysis results indicated that the average annual cost of ITN delivery (2005 USD 3.98) was very attractive when compared with past ITN delivery studies at different scales. Financing was largely from donor sources though the Eritrean government and net users also contributed funding. The intervention's cost-effectiveness was in a highly attractive range for sub-Saharan Africa. The cost per DALY averted was USD 13 – 44. The cost per death averted was USD 438–1449. Distribution of nets coincided with significant increases in coverage and usage of nets nationwide, approaching or exceeding international targets in some areas.

**Conclusion:**

ITNs can be cost-effectively delivered at a large scale in sub-Saharan Africa through a distribution system that is highly integrated into the health system. Operating and sustaining such a system still requires strong donor funding and support as well as a functional and extensive system of health facilities and community health workers already in place.

## Background

Vector control generally and insecticide-treated nets (ITNs) specifically have been identified as effective and cost effective methods for the prevention of malaria related mortality and morbidity in sub-Saharan Africa (SSA) [1]. Despite the vast expansion of vector control implementation, relatively few studies have examined the key economic characteristics of programmes operating on a large scale [2-5]. Considerable debate still remains as to the most appropriate strategy for delivering ITNs on a national scale [6]. Further, the relative merits of ITNs versus Indoor Residual Spraying (IRS) are also insufficiently documented. This study provides data on the operations, costs and estimates of the cost effectiveness of the national ITN programme in Eritrea. This analysis is part of larger multi-country analysis of the main strategies used by ITN and IRS programmes in SSA [7].

**Figure 1:**
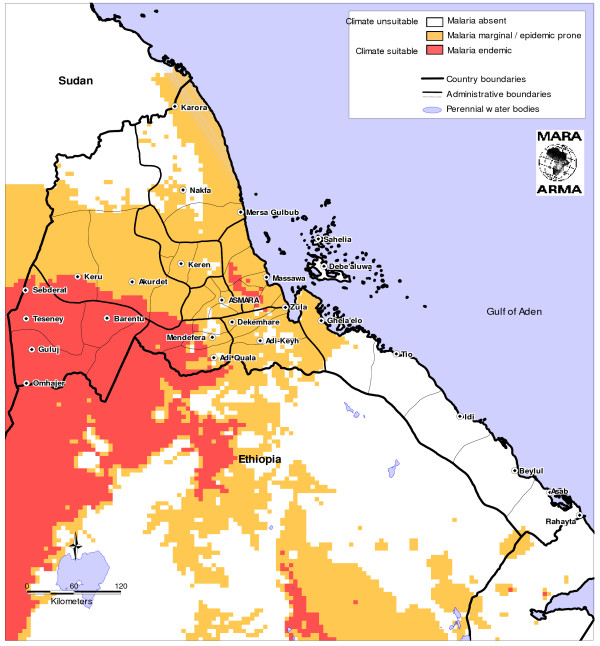
**Climatic suitability for malaria transmission in Eritrea (source: Mapping Malaria Risk in Africa, )**.

Eritrea is divided into three zones of malaria risk based on epidemiology and geography. In the western lowlands, where the malaria burden is most severe, transmission is highly seasonal and most intense along water bodies or irrigation projects. The transmission season lasts from September until November. In the highlands there is generally little risk of malaria transmission, but due to the low immunity of the population there is a risk of epidemic malaria. Finally, in the coastal plains along the Red Sea, highly seasonal malaria transmission occurs which peaks between January and March, though transmission in this zone is generally low due to low levels of precipitation. A recent study estimated that two-thirds of the Eritrean population are considered at risk of malaria, either epidemic or endemic [8], and the Mapping Malaria Risk in Africa (MARA) project estimates that approximately 92% of the population is at risk for malaria, with approximately 51% at risk of endemic malaria [9](Figure 1). The population of Eritrea in the year 2005 was estimated to be 4.3 million [9]. Of this number, approximately 760,000 were children under five years of age. Using an assumption that 5% of the population is made up of currently pregnant women, there are approximately 215,000 such women in the country, while a total population of 2.9 million lives in malaria-endemic areas.

The Eritrean ITN programme is part of a broader malaria control programme which includes environmental modification, larviciding, IRS and the provision of prompt and effective treatment. The mix of interventions used varies depending on the area of the country, with IRS being used more extensively in the highest burden areas and environmental management and larviciding used in all malarious regions, but most extensively in moderate to low burden areas [10-12]. The ITN programme currently utilizes free distribution of ITNs to high-risk groups through ante-natal care (ANC) clinics, and to the general population in malarious areas through community health agents (CHA) and local administrations. This has provided a "catch-up"strategy in Eritrea, increasing ITN coverage rapidly among households and vulnerable groups by providing a large number of free and low-cost nets. Recent coverage data are shown in Table 1. It has also maintained a more continuous pipeline and thereby helped to "keep-up" coverage in the longer term as well [10,13,14]. Generally the programme has achieved relatively high ITN coverage and usage results including reaching household possession over 80%, and meeting Abuja Target usage levels in some regions and high coverage and usage overall [10,14,15]. Several studies have found that ITN distribution has been effective in reducing the incidence of clinical malaria in Eritrea [10,12,16]. Malaria specific mortality and case fatality rates also fell during the period of dramatic increases in ITN coverage in Eritrea [10,17-21]. As of 2008, the Eritrean programme still represents the only national-scale ITN interventions in SSA that relies almost entirely on routine public sector implementation. While overall results of this study have been published in a multi-country comparative study [7], this paper provides an opportunity (1) to show the unique features of the Eritrea programme, (2) present in much more detail the data used for the comparative study, and (3) illustrate the ways in which average cost-effectiveness results can be confounded by issues of scale and timing of measurement.

**Table 1:** Net coverage and usage in Eritrea according to latest available statistics.

	2002 DHS*		2002 DHS**		2003 NMCP/Tulane**		2004 NMCP/RBM***	
	**Any net**	**ITN**	**Any net**	**ITN**	**Any net**	**ITN**	**Any net**	**ITN**

**Children under nets last night**								

**All**	12.1%	4.2%		7.3%		76.6%		58.6%

**Urban**	14.3%	4.8%						

**Rural**	11.0%	4.0%						

								

**Pregnant women under nets last night**								

**All**	6.6%	2.9%		4.7%		52.6%		50.4%

**Urban**	8.7%	4.5%						

**Rural**	5.5%	2.1%						

								

**Household possession**								

**All**	33.8%		47.8%		92.3%	82.9%	79%	62%

**Urban**	28.3%							

**Rural**	37.3%							

NMCP = National Malaria Control Programme; RBM = Roll Back Malaria.

*ITN defined as a net treated within the last 6 months.

**ITN defined as any net treated within the last 12 months, surveys restricted to Gash-Barka and Anseba zobas for purposes of comparison. DHS survey is dry season, while the Tulane survey was conducted during the rainy season.

***ITN defined as any net treated within the last 6 months, survey conducted in Gash-Barka, Anseba, Debub and the Northern Red Sea zobas, where malaria control measures are active (data from [10]).

## Methods

### ITN programme: operational description

ITN distribution began in 1995 as a pilot project in Gash-Barka, in the western lowlands. ITNs were delivered through health facilities and local administrations, with assistance from CHAs. The programme was gradually scaled up from a few villages to cover all malarious areas of the country. Families were charged 30 Nakfa (USD 2–3) per ITN before the 2003 distribution. Local administrations could make exceptions for those families deemed too poor to pay. In 2001, free distribution to women at ANC and maternal and child health (MCH) clinics began, and from 2003, nets were also given freely to the general population living in malarious areas. Since 2001, over 900,000 ITNs have been delivered and two million re-treatments have been conducted through community campaigns and health facilities [17-21].

Re-impregnation campaigns are conducted once a year, one to two months prior to the malaria transmission season, at health facilities and community re-treatment points. Supervision is ensured by health facility staff with the support of CHAs and with the collaboration of community members.

**Figure 2:**
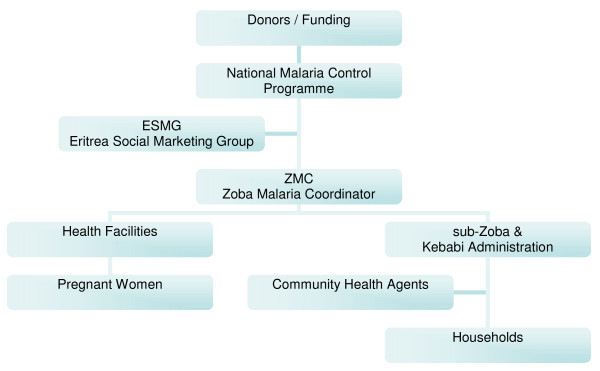
**Structure of the Eritrean national ITN programme**.

Administratively the country is divided into six regions, called Zobas, and further into sub-Zobas (districts) and Kebabis (agglomerations of ~3–10 villages). Four of the Zobas are considered to be at significant malaria risk, with the highest risk Zobas being Debub and Gash Barka and the lowest risk in the Northern Red Sea Zoba [15]. Operationally, the ITN programme functions similarly in all malarious regions with the caveat that some areas considered at higher risk were targeted for earlier distributions of ITNs. A full description of the Eritrean ITN programme is available in an unpublished report [22]. The structure of the programme is shown schematically in Figure 2.

The ITN distribution system in Eritrea was highly successful in raising the level of coverage to Abuja target levels and survey evidence that the country had met the 60% coverage targets set under that agreement within specific regions became available in 2003 (See Table 1) [14]. Given that success, the National Malaria Control Programme (NMCP) set higher targets at its annual meeting in Tesseney in 2004 (80% household ownership and pregnant women/under five usage by 2006) [23]. These targets are in line with current RBM international policy [1]. Unfortunately, there is no available survey data to provide evidence as to the expansion or maintenance of the success of the Eritrea programme after 2004, however, a NMCP/Tulane survey undertaken in 2003 showed that when ITNs were defined as nets treated within 12 months nearly 80% of households in the most highly malarious areas of the country already owned ITNs (Table 1 shows selected results of ITN coverage and usage surveys in Eritrea). Distribution of nets in Eritrea has continued since the last survey was conducted.

### Time frame and perspective for costing exercise

Costs and outputs collected for this study cover a time period which extended from 2001, when nets were first given freely though ANC facilities, through the end of 2005, after three full years of community-wide distribution. This time interval covered a period of large scale up in ITN coverage, although it did not include the initial expansion of the programme to all malarious areas of the country [13,14]. Hence, it was not possible to include the start-up costs of the programme.

Attempts were made to follow existing guidelines for the cost evaluation of ITN programmes, as well as to use methodologies similar to past studies in order to maximize comparability [24,3,25]. On this basis, all costing methods were standardized with six other national programme assessments that were conducted concurrently [7,22].

### Types of costs included

The study generally used the provider perspective. Travel or time costs to users, or other household-level costs or cost savings have not been measured or included, however, user charges from the early years of the program were included when calculating sources of financing. All direct and shared costs of the ITN programme to the provider were included, including those of the commodities and their delivery, health promotion, and social mobilization, as listed in Table 2. Costs represent the marginal cost of adding Eritrea's ITN intervention onto an existing malaria control programme and health system. These expenses include the cost of training health workers in ITN use and re-treatment, health promotion and social mobilization, use of facilities, transportation, payment of personnel, and the commodities themselves. Costs required to build and train a network of Community Health Agents (CHAs) or to develop a network of health facilities have not been included.

**Table 2:** Types of costs included in the Eritrea analysis.

**Capital**	Buildings
	Vehicles
	Equipment and furniture
	ITNs
**Recurrent**	Insecticide
	Personnel
	Training and meetings
	Fuel/maintenance of vehicles
	Office/warehouse rental
	Advertising and promotion
	Supplies/overheads
	Patients' expenditures (excluding cost of ITN)
	Management cost
	Basic evaluation and monitoring (excluding specific research cost)

### Cost collection

Costs were collected retrospectively from financial and operational records kept by the NMCP, zonal offices in the four main malarious zobas of Eritrea, the Eritrean Social Marketing Group (ESMG), the World Bank-funded HAMSET project, and the Global Fund to Fight AIDS, TB and Malaria (GFATM). Cost and activity information collection occurred during two periods, from May to June 2005 and from May to June 2006. Costs were collected by examining agencies' financial records, including budgets, expenditure records, reports, receipts and invoices. In addition, cost and activity information were collected through stakeholder interviews and direct observation where costs were not reflected in the financial records.

Where possible, the ingredients approach was used, meaning that unit activities were first determined and then a unit cost was determined for these activities, allowing for the establishment of a total cost. Where this approach was not possible, either because the information was deemed too sensitive or was not available in adequate detail, aggregated expenditure was generally used.

Resource use was valued at three levels: centrally (within the MOH and the NMCP), at the Zoba level, and peripherally including health workers, health facilities, and CHAs. Direct costs to Kebabi administrators have not been included since they are believed to be small and no reliable information about these activities could be acquired within the framework of this study.

Resources were valued based on the reported expenditures or budgets, and in the case of shared personnel, on salary plus any fringe benefits. Capital goods were valued based on their procurement costs or alternatively, in the case of building rents, on an average market value of similar properties as reported by the Eritrean government.

### Cost classification and adjustments

Costs have been divided into *capital *and *recurrent *costs based mainly on the lifetime of the goods being purchased. As stated above, it was not possible to collect start-up costs. Because of the long and gradual build-up to a full national scale programme, the start-up costs were likely to be large but also difficult to define as they were spread over a long time period and involved pilot programmes which would not be replicated if the strategy were used elsewhere. Due to the long development period, these costs would have little effect on the current running costs of the programme, but failure to capture them reduces the stated costs. Capital costs have been discounted in the economic analysis using lifetimes and discount rates determined through stakeholder interviews, expert information, and past literature. Varying discount rates and lifetimes were examined in the sensitivity analysis. Both financial and economic analyses were conducted, in order to show (1) what the actual expenses of running a programme would be, as well as (2) the level of all resources used. In the financial analysis, taxes on nets and insecticide have been included, while in the economic analysis, they have been treated as transfer payments and have therefore been excluded. In the financial analysis, capital costs have not been discounted and are instead incurred in full at the time of the purchase.

Costs were collected in either Eritrean Nakfa or United States Dollars (USD). Costs collected in Nakfa were converted to USD based on official yearly average exchange rates for the period during which the costs were incurred, and all costs were adjusted for inflation to 2005 prices using the US Gross Domestic Product deflator produced by the US Bureau for Economic Analysis [26].

### Outputs

Generally, two main output measures were used: (1) *number of nets delivered *and (2) *number of re-treatments performed*. Both figures were reported by the NMCP annually, and reflect nets and re-treatments delivered at the primary health care facility level or community level. In the years 2001 and 2002, care was taken to disaggregate nets delivered via ANC clinics and community mechanisms, as nets delivered through community mechanisms involved a user fee, while nets delivered through ANC clinics did not. The number of re-treatments performed during annual re-treatment campaigns were reported by the Eritrea NMCP [17-21].

The two output indicators above were used to calculate a third combined output measure: *treated net years (TNY)*, which blends the results of both outputs by assuming that either a re-treatment or a new ITN provides one year of full protection for any individual using that net. This combined indicator is useful because it allows the inclusion of re-treatments of existing nets as part of the outcome measure, while the calculation of the cost per ITN delivered does not. Though some nets in the year 2005 were LLINs, all nets have been treated as conventional nets for the calculation of outputs in the base scenario of this analysis. The number of TNYs delivered through an LLIN will be higher – approximately 3–5 years per net. In the sensitivity analysis, outputs were also calculated using the assumption that all nets delivered were LLINs.

### Cost-effectiveness calculations

Two separate cost-effectiveness ratios were measured: (1) *cost per child death averted *and (1) *cost per disability-adjusted life year (DALY) averted*. Each of these ratios has been calculated using both the cost and effects of ITNs delivered and TNYs achieved. Only the effects of ITN use and possession by children were included in either calculation, thus ignoring potential benefits to older individuals. While this calculation underestimates the morbidity burden averted by ITN use, it would be difficult to include other effects, which are not well quantified at present [27]. It is also consistent with the fact that the DALY burden in malaria-endemic countries is largely driven by child deaths [28]. The effects of untreated nets were excluded in the analysis because (1) it would have been difficult to obtain information on both numbers of such nets and their effect, and (2) the effects of long-lasting insecticidal nets (LLIN) were included in the sensitivity analysis, a more relevant scenario for the future.

Cost results were coupled with standard health impact indicators derived from (1) the Cochrane review on ITNs [29], and (2) WHO methodology for the calculation of DALYs averted [30]. Estimates from the Cochrane review show that approximately 5.5 child deaths are prevented for every 1,000 children who are protected by a net for one year, or approximately 33 DALYs per death if all deaths are treated as infant deaths. This output has been combined with both cost outputs (cost per ITN delivered and cost per TNY delivered) to produce the final cost-effectiveness ratios.

### Base scenario

In this analysis, the base case scenario relies on the following set of assumptions. A discount rate of 3% has been applied to both DALYs (see below) and to economic capital costs. Shared costs have been attributed to the interventions based on the actual value of the indicator chosen to allocate them. Nets have been assumed to last for 3 years and, as noted above, treated nets and re-treatments are set to provide 1 year of protection each. Fifty percent of the nets delivered were assumed to be used by children and only one child was assumed to sleep under each of these nets. The cost of nets was based on the commodity, insurance and freight (c.i.f.) price of the nets; for the portion of nets delivered with user fees through community mechanisms the user fee was included for the purpose of calculating sources of funding (the proportion of nets delivered this way was assumed to be 50% in the early years of the programme). Clearly, these are conservative estimates, mainly chosen in order to make a cross-country comparison easier and more meaningful [7].

## Results and Discussion

**Figure 3:**
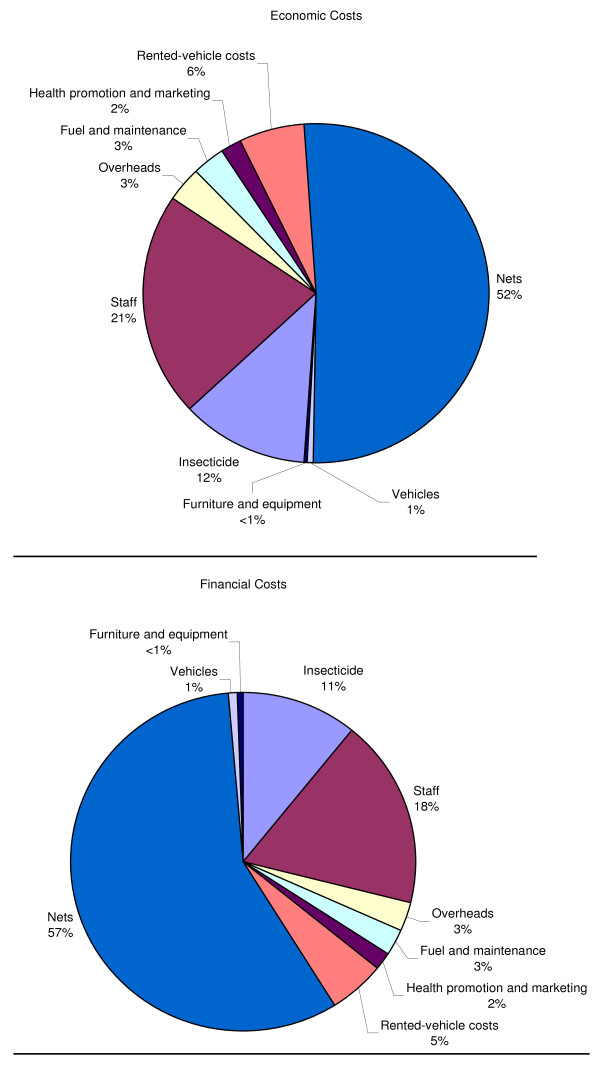
**Economic and financial cost composition of the Eritrean ITN programme**.

The Eritrean national ITN programme delivered over 900,000 ITNs and over two million re-treatments during the period 2001–2005, at a total economic cost of USD 3.7 million, or a financial cost of just under USD 4.4 million USD (Table 3). The cost composition (Table 3 and Figure 3) of the programme was quite similar to that of other programmes which have been examined [7]. In economic terms, about 50% of the costs of the programme were capital costs, which were accounted for almost entirely by the cost of the nets. The commodity costs (nets and insecticide) represented 63% of the total economic cost of the programme. Other major costs were staff (21%), and vehicle costs (approximately 7%).

**Table 3:** Cost composition of the Eritrean national ITN programme (2005 USD).

**Line item**	**Economic**		**Financial**	
	**Total cost**	**%**	**Total cost**	**%**

**Recurrent**				

Insecticide	442,213	12%	473,698	11%
Staff	787,638	21%	787,638	18%
Overheads	125,309	3%	125,309	3%
Fuel and maintenance	111,284	3%	111,284	3%
Health promotion and marketing	73,850	2%	73,850	2%
Rented-vehicle costs	229,984	6%	229,984	5%
				
**Subtotal recurrent**	**1,770,278**	**48%**	**1,801,764**	**41%**

				

**Capital**				

Nets	1,901,729	51%	2,519,343	58%
Vehicles	18,527	1%	39,294	1%
Furniture and equipment	7,704	<1%	19,614	<1%
				
**Subtotal capital**	**1,927,960**	**52%**	**2,578,251**	**59%**

**Grand total**	**3,698,238**	**100%**	**4,380,015**	**100%**

In financial terms, the cost composition of the programme appeared somewhat differently. The largest differences were due to the removal of annualization for nets, which significantly increased their costs in any given year, as well as their share of the total cost of the programme. Secondly, there was a small increase in the costs of nets and insecticide due to the inclusion of import taxes. Other capital costs also increased significantly, but their overall contribution to the cost of the programme was generally low, so these increases had little effect on overall spending patterns.

Table 4 summarizes the results of the costing analysis in terms of cost per ITN and TNY delivered. The results indicate an economic cost of approximately USD 3.98 per ITN delivered over the period 2001–2005 and a financial cost of USD 4.72. The cost per ITN delivered excluding the commodity cost (ITNs and re-treatments) was about USD 1.50. Costs per TNY were significantly lower due to the large numbers of re-treatments conducted during annual re-treatment campaigns, which increased the denominator in the TNY ratio significantly. When the commodity costs were excluded, the Eritrean programme delivered a year of protection for an approximate economic cost of USD 0.44.

**Table 4:** Annual costs per ITN and Treated Net-Year (TNY) delivered in Eritrea (2005 USD).

**Year**	**2001**	**2002**	**2003**	**2004**	**2005**	**Average/Total**
**Financial cost (per ITN)**	4.98	3.56	3.87	4.43	9.39	**4.72**
**Economic cost (per ITN)**	3.15	2.47	4.14	4.07	8.51	**3.98**
**Economic cost (per ITN) less commodities**	2.02	1.03	1.23	1.23	2.68	**1.46**
**Financial cost (per TNY)**	2.55	1.95	1.06	1.25	1.20	**1.43**
**Economic cost (per TNY)**	1.61	1.35	1.14	1.15	1.09	**1.21**
**Economic cost (per TNY) less commodities**	1.03	0.57	0.34	0.35	0.34	**0.44**
**ITNs delivered**	141,766	276,038	187,709	214,752	108,011	**928,276**
**Re-treatments**	135,290	227,750	497,117	544,464	734,154	**2,138,775**

The Eritrean national ITN programme was largely funded through donor contributions. However, in the early stages of the Eritrean national ITN programme (before 2003), user charges were collected for nets delivered through community mechanisms. Unfortunately, it was not possible to determine the specific number of nets for which user charges were applied. As these charges were used to fund the programme, an assumption that 50% of nets delivered before 2003 included user fees when estimating the contribution to the program from each sector was used. In this scenario, donors contributed 69% of funding, users contributed 14% and the MoH contributed 17%. As the practice of charging user fees was discontinued after 2002, the programme relied much more heavily on donor and governmental financing from 2003 onward. Investment from the government of Eritrea was largely in terms of salaries, building space, and vehicles.

### Returns to scale

When the costs were disaggregated by year (Table 4), several interesting findings emerged. One was that the cost of ITN delivery did not fall over time, partly because there was no trend toward an increase in scale over the time period analysed. In fact, the number of nets delivered fluctuated over time and the figures were substantially lower in 2005 compared to previous years.

Evidence of the increasing returns to scale is more apparent in these results due to the large increases in TNYs delivered as compared to the ITNs delivered. However, some of this effect was mitigated after 2003 by rising costs for net procurement. If, however, the analysis took into account the fact that some of the nets delivered in 2005 were LLINs and thus provide more than one TNY, the costs per TNY would have fallen despite the real increase in the cost per ITN delivered (*data not shown*).

**Figure 4:**
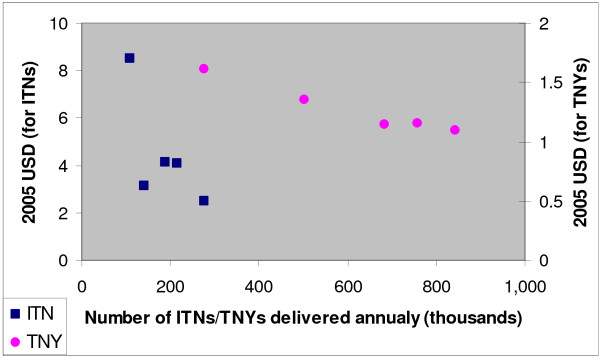
**Annual economic costs vs. total output (period 2001–2005)**. Annual economic cost per ITN or Treated Net-Year (TNY) delivered vs. total annual number of ITNs or TNYs delivered in Eritrea (period 2001–2005).

Figure 4 and Table 5 illustrate the scale efficiency savings due to increases in the number of TNYs of protection and the numbers of ITNs delivered. In Eritrea's programme it is relatively straightforward to segregate procurement costs from distribution costs, and the former may be a good proxy for savings due to changes in the price of the inputs versus the latter. This can be used to examine changes due to efficiencies of delivery (or economies of scale in distribution). Separating these costs and analyzing their annual fluctuations shows that the programme achieved a savings of over 60% in terms of TNY distribution by its final year. These savings are largely attributable to the increasing share of re-treatments in the input mix, which significantly reduced the average cost of delivering one TNY of protection. The programme also showed approximately a 30% reduction in the total unit cost per TNY over the study period, indicating significant returns to scale in the system, despite procurement costs which increased. These results argue strongly for operating on a larger scale, as well as for the desirability of delivering re-treatments for nets when the use of LLINs is not generalized. Evidence that providing re-treatments to existing nets can be more cost-effective than delivering new nets has been developed previously in the literature [31]. Figure 3 shows how with increasing scale, the costs of distribution fell significantly in Eritrea both for TNYs and ITN delivery. As there is no trend of increasing scale over time in the Eritrean programme, the figure shows the cost versus annual delivery of nets and helps to better illustrate the scale efficiencies in the system.

**Table 5:** Scale efficiency savings in the Eritrean national ITN programme

**Year**	**2001**	**2002**	**2003**	**2004**	**2005**	**Total/average**
**Output growth (actual)**		113,366	90,519	37,195	311,502	1,665,025
**Output growth (%)**		82%	36%	11%	82%	53%

***Total cost***						
**TNY unit cost**	1.61	1.35	1.14	1.15	1.09	1.21
**TNY unit cost savings**		0.26	0.22	-0.02	0.06	0.52
**TNY unit cost savings (%)**		16%	16%	-1%	5%	32%

***Procurement only***						
**TNY Unit Cost**	0.58	0.79	0.80	0.80	0.75	0.76
**TNY Unit Cost Savings**		-0.21	-0.01	-0.01	0.05	-0.17

**TNY Unit Cost Savings (%)**		-36%	-1%	-1%	7%	-29%

***Distribution only***						
**TNY Unit Cost**	1.03	0.57	0.34	0.35	0.34	0.44
**TNY Unit Cost Savings**		0.47	0.23	-0.01	0.00	0.69
**TNY Unit Cost Savings (%)**		45%	40%	-3%	1%	67%

TNY = Treated Net Year

However, it should be pointed out that while there might be a significant amount of spare capacity within the Eritrean ITN programme, at least in low net delivery years, the integrated nature of the programme means that this spare capacity is likely to have been used for other productive activities.

### Cost-effectiveness

Cost-effectiveness calculations began with the base case cost analysis and were derived using either cost per ITN or cost per TNY, as shown in Table 4. Table 6 shows the predicted health impacts of the intervention in a base scenario analysis. Using the total TNY measure of protection, it can be seen that significantly more deaths and thus DALYs were predicted to have been averted by the intervention due to the addition of protection through re-treatment delivery. By contrast, when only conventional ITNs, without re-treatment, were included in the calculation, the predicted health impact was much lower. These data illustrate again the urgent need to introduce LLINs instead of conventional nets.

**Table 6:** Estimated cost-effectiveness of the Eritrean national ITN programme (2005 USD).

	**Using cost per ITN**	**Using cost per TNY**
**Deaths averted (2001–2005)**	2,553	8,434

**DALYs averted (2001–2005)**	84,241	278,335

**Cost per death averted (USD)**	1,449	438

**Cost per DALY averted (USD)**	44	13

TNY = Treated Net-Year DALY = Disability-Adjusted Life Year

Table 6 also illustrates the overall cost-effectiveness ratios that were derived in the base analysis. Overall, cost per death and DALY averted were generally low in terms of previously published WHO criteria for assessing interventions and research [32], but vary markedly depended on the output measure used. As expected, TNYs yielded significantly lower costs per death averted and DALY averted due to the large number of re-treatments provided by the programme.

### Sensitivity analysis

Sensitivity analysis was conducted to examine the effects of key assumptions on the cost per output and on cost-effectiveness ratios. The parameters examined included: lifetime of nets, discount rate, allocation of shared costs, the costs of nets and insecticide, and two scenarios corresponding to the use of Long-Lasting Insecticidal Nets (LLINs). The parameters have been varied to the extremes of their potential ranges, as determined by reviews of the literature or expert opinion.

Sensitivity analysis was also applied to the main assumptions about the connection between the cost output ratios and the cost-effectiveness calculations. Parameters examined included the usage rates of the nets delivered, the percentage of nets used by children, the length of time during which a net or re-treatment offers protection, including the extended protection offered by LLINs, and the cost-to-output parameters which affect the overall cost of delivering a net or re-treatment.

The LLIN scenarios were calculated by assuming a nominal price and lifetime for types of LLINs: USD 5.00 and 3 years of protection or USD 7.00 and 5 years of protection. These scenarios were expected to bracket the cost-effectiveness ratios which would have been generated by assuming that some protection was offered by untreated conventional nets, an effect which was ignored in our base scenario. Table 7 shows the results of a sensitivity analysis for the cost per ITN and TNY delivered. Table 8 shows the results of the analysis for the other outputs and outcomes: deaths averted and DALYs averted.

**Table 7:** Results of sensitivity analysis: cost per ITN and Treated Net Year (TNY) delivered in Eritrea (2005 USD).

**Parameter**	**Values assumed**	**Range (USD) per ITN**	**Range (USD) per TNY**
**Discount rate**	1–10%	3.90–4.27	1.18–1.29

**Cost of nets**	1.50–5.00–7.00	3.62–6.88–8.75	1.09–2.08–2.65

**Lifetime of nets**	1 year – 5 years	4.49–3.47	1.36–1.05

**Cost of insecticide/liter**	6.00–12.00	3.80–4.10	1.15–1.24

**Shared costs**	0–100%	3.29–10.30	1.00–3.12

LLIN use*	Base – 3 years, USD 5 – 5 years, USD 7	3.98–7.75–10.08 (NA – 7.28 – 9.60)	1.43–1.46–1.38 (NA – 2.43 – 1.92)

**Length of protection offered by ITN or re-treatment**	3 months – 6 months – 1 year	N/A	5.73–2.87–1.43

LLIN = Long-Lasting Insecticidal Net.

*numbers in brackets represent exclusion of re-treatment commodity costs and benefits

**Table 8:** Results of sensitivity analysis: cost per death and Disability-Adjusted Life Year (DALY) averted in Eritrea (2005 USD).

**Parameter**	**Values assumed**	**ITN range (USD) per death averted**	**TNY range (USD) per death averted**	**ITN range (USD) per DALY averted**	**TNY range (USD) per DALY averted**
**Discount rate**	1–10%	1,420–1,554	430–470	43–47	13–14
**Cost of nets**	1.50–5.00–7.00	1,315–2,503–3,183	398–758–963	40–76–96	12–23–29
**Lifetime of nets**	1 year – 5 years	1,634–1,263	495–382	50–38	15–12
**Cost of insecticide/liter**	6.00–12.00	1,383–1,490	419–451	42–45	13–14
**Shared costs**	0–100%	1,196–3,745	362–1,134	36–113	11–34
**Length of protection offered by ITN or re-treatment**	3 months – 6 months – 1 year	5,795–2,897–1,449	1,754–877–438	176–88–44	53–27–13
**Usage of nets**	25–50–100%	2,897–1,449–724	877–438–219	88–44–22	27–13–7
**Children protected per net**	1–2	1,449–724	438–219	44–22	13–7

LLIN use (TNY)*	Base – 3 years, USD 5 – 5 years, USD 7	438–531–502 (NA – 882 – 698)		13–16–15 (NA – 27 – 21)	

TNY = Treated Net Year; LLIN = Long-Lasting Insecticidal Net.

*numbers in brackets represent exclusion of re-treatment commodity costs and benefits

The overall range of results for the cost per ITN delivered is quite wide, ranging from USD 3.29 to 10.30, indicating that there are significant uncertainties in the cost measurements. However, there are several reasons to believe that the actual variance is less than that suggested by the extremes of the sensitivity analysis. The upper values only exceed approximately USD 5.00 in the case of two variables: the cost of the nets and the allocation of shared costs. In terms of the cost of nets, USD 7 per net is an unlikely figure since the Eritrean programme was able to procure LLINs in 2005 for an average cost of USD 4.85. The other variable which gives rise to the most extreme value in the sensitivity analysis is the allocation of shared costs. In most cases, the personnel involved in the Eritrean ITN programme are involved with ITN activities for only a portion of the year, and even during that time, they are typically not fully dedicated to the programme. Most allocation measures, which were based on either the days of per diem paid to employees, or the amount of space used in offices and buildings, indicated that less than one-third of a given shared resource was being used for ITNs.

For the calculation of deaths averted and DALYs averted, the assumptions regarding (1) the length of protection provided by an ITN or re-treatment, and (2) assumptions concerning the usage of these nets among children had a dramatic impact on the results. While published information suggests that usage rates for children in net-owning households were high (on the order of 80%, meaning that 80% of households with nets had at least some children using them), the link between the actual number of nets delivered by the programme and usage is less certain [15]. As a matter of course, some of the nets will be lost, damaged or misused. Hence, even if usage among households owning nets is high, the actual relationship between household ownership and nets delivered will yield lower rates of usage per net delivered. It was not possible to explicitly quantify this effect. However, usage rates were varied to include expected wastage rates in the sensitivity analysis (from 25% of nets used by children to 100%). Our assumptions were generally conservative, but it is clear that if usage of nets was worse and/or wastage higher, the cost effectiveness results presented here would overestimate the values presented for Eritrea. Additionally it should be noted that while we calculated health impacts in a manner intended to be consistent with the other studies in a large multi-country comparison, this scenario is reflective of a highly endemic malaria situation [7]. In a country with malaria epidemiology as varied as Eritrea's these results may not be reflective of the true epidemiological impact of the intervention in all settings.

When the scenarios using different LLIN assumptions were calculated, the cost-effectiveness ratios in all cases approached the result obtained by delivering inexpensive conventional nets and large numbers of re-treatments through campaigns. As it was not possible to disaggregate all re-treatment campaign costs from the analysis, it would be expected that the true LLIN cost-effectiveness ratios would improve slightly compared to those shown here.

## Conclusion

Under the base case scenario the cost per DALY averted was as low as 13 USD. Considering that this is a conservative scenario, the result is clearly within the acceptable range for interventions in low-income countries as defined in previous WHO publications [32]. The results of the sensitivity analysis clearly showed that some parameters, such as the usage of the nets, their cost, and the length of protection delivered had important effects on cost per output and cost-effectiveness. Scenarios which incorporated LLINs showed only small changes in cost-effectiveness over the delivery of conventional nets and re-treatments but did so despite substantial increases in the upfront cost of ITN procurement. Since the use of LLINs would eliminate the need for re-treatment campaigns it appears that a full shift to LLIN technology would be beneficial in Eritrea and this has been adopted as the current national policy.

The Eritrean National ITN programme has increased ITN coverage dramatically to the point that it has essentially met the Abuja targets for ITN coverage in vulnerable groups within at least some areas of the country [10,14]. The new target of 80% protection in high-risk groups [1,23] should also be within reach of the programme. Two studies used regression analysis to demonstrate that ITNs had a measurable effect on clinical malaria incidence [10,12]. The cost-effectiveness results presented here indicate that Eritrea has achieved this goal in an efficient manner and that it is possible to distribute ITNs at no charge to users through a system fully integrated into the existing health system. This is currently a unique model in SSA and worth investigating for other countries. It must be stressed, however, that such a system relies (1) on an adequately functioning health system, and (2) on a good outreach system, in the case of Eritrea in the form of CHAs. If these two conditions are not met, such a public sector approach might not be an effective way to distribute ITNs.

Similarly to the four other ITN programmes reviewed in a larger comparative study [7], the Eritrean ITN programme is largely dependent on external financing, underscoring the importance of reliable and predictable funding for vector control activities. Ultimately, the Eritrean programme has been both successful in providing wide reaching high ITN coverage and sustaining it over a period of some years. Furthermore it has done so in a cost-effective manner, thus providing an example for other SSA countries, whether they still need "catch-up" ITN activities or need to "keep-up" already high ITN coverage.

## Competing interests

MZ and TG are employed by the NMCP of Eritrea. All other authors declare that they have no competing interests.

## Authors' contributions

JY assisted in the design of the research and the data collection, and he conducted the data analysis and drafted the manuscript. MZ and TG collected data as well as critically reviewed the manuscript. FTassisted in the conception, design, data analysis and in the drafting and review of the manuscript. CL conceived and designed the research and he assisted in the drafting and review of the manuscript.
